# Bacterial biodiversity and metagenomic study of dadih, traditional fermented buffalo milk from Kampar district, Riau, Indonesia

**DOI:** 10.5455/javar.2025.l935

**Published:** 2025-08-18

**Authors:** Evy Rossi, Usman Pato, Dewi Fortuna Ayu, Sri Melia, Ade Sukma, Rahmayuni Rahmayuni, Annisa Nazifa Salman

**Affiliations:** 1 Department of Agricultural Technology, Faculty of Agriculture, Universitas Riau, Pekanbaru, Indonesia; 2 Department of Animal Product Technology, Faculty of Animal Science, Universitas Andalas, Padang, Indonesia

**Keywords:** Dadih, buffalo milk, fermentation, metagenomic, next-generation sequencing

## Abstract

**Objective:**

This study aimed to investigate the metagenomic and microbial diversity of dadih in Kampar District, Riau, Indonesia.

**Materials and Methods:**

The dadih samples were collected from dadih producers in three villages, namely Limau Manis (LM), Rumbio (RB), and Muaro Jalai (MJ). DNA samples were extracted and sequenced through Oxford Nanopore Technology (ONT), operated by MinKNOW software version 23.04.5. Library preparations were conducted using kits from ONT.

**Results:**

The next-generation sequencing analysis on three dadih from Kampar identified two bacterial phyla, Bacillota and Pseudomonadota. Furthermore, there was a slight variation in dadih's microbiota composition between LM, RB, and MJ. The Bacillota phylum dominated the dadih microbiota in LM and RB villages, with a relative abundance of 60%–80%. The dadih from MJ was dominated by the phylum Pseudomonadota, which reached 55%. The dominant species found in all three dadih was *Lactococcus lactis*, with an abundance of 53.80, 80.80, and 40.31% for dadih LM, RB, and MJ, respectively.

**Conclusion:**

Dadih MJ had the highest Simpson's value (~0.8), showing a relatively even abundance of species in the sample. Furthermore, dadih LM had a high Simpson's value (~0.75), indicating similar conditions to dadih MJ. Dadih RB had the lowest Simpson's value (~0.4), confirming that the microbiota in the sample tends to be dominated by certain species with a less even distribution.

## Introduction

For thousands of years, people have consumed fermented foods worldwide, which are essential to life. Various fermented foods have existed throughout civilization, with methods derived from the resources available in a region [1]. The primary sources of microorganisms used in food fermentation are natural starters [2, 3] and modified starter cultures for yogurt or milk fermentation [4, 5], Chinese wine [6], and sausage [7]. The microbial community makeup in conventional food fermentation systems is typically complex and varied [8].

Traditionally, fermentation was practiced as a preservation method to improve microbiological safety while extending the shelf life of food products [8] to eliminate or suppress pathogens when cold storage methods had not been invented [9]. Various kinds of fermented products are found in different countries, such as kimchi from Korea [10], da-jiang from China [11], and dadih (dadio) from Indonesia [12]. These products contain many lactic acid bacteria (LAB), particularly probiotics, which have been proven to protect against pathogenic bacteria [13], treat digestive disorders in the intestine [14], reduce lactose intolerance [15], maintain serum cholesterol levels [16], have anti-carcinogenic activity [17], lower triglycerides [18], act as a bio-preservative [19, 20], and enhance immune function [21].

Increased public awareness of health has led to a high demand for functional foods. One of Riau, Indonesia's indigenous functional foods, is dadih, a fermented buffalo milk product produced in Kampar District. Kampar dadih holds enormous potential for the local community, who believe it can nourish the body, prevent diseases like hypertension and hypercholesterolemia, and maintain heart health. The potential of Kampar dadih has not been fully explored compared to dadih from West Sumatra, which is widely known as a functional food. Dadih has antimicrobial activity [22] and antibiotic resistance [23], produces γ-aminobutyric acid (GABA) [24], and inhibits pathogens' colonization in the digestive tract [25]. A previous study by Surono [26] reported that 10 strains of LAB from dadih showed acidity and tolerance to bile acids *in vitro*, indicating their potential as antimutagenic and hypocholesterolemic agents. The LAB probiotics derived from dadih have significantly reduced total cholesterol, serum low-density lipoprotein (LDL) cholesterol, and bile acids [27].

The tools and raw materials used to make Kampar dadih differ from West Sumatra dadih. Kampar dadih uses microbiota in *buluh *(small bamboo tubes), while West Sumatra dadih is fermented in *betung* (giant bamboo). The variation in fermentation containers causes the two different dadih origins to have a diversity of microbiota to produce separate functional properties [28, 29]. The fermentation of dadih includes a variety of native LAB, which affects the composition of final products [30].

Understanding the microbiota composition and population dynamics in dadih is essential for optimizing fermentation and enhancing product quality. Traditional culture methods often miss non-culturable species, making molecular-based methods like next-generation sequencing (NGS) crucial for metagenomic analysis. The NGS provides detailed insights into microbial diversity at both species and genus levels, especially for rare microorganisms. Sequencing *16S ribosomal RNA* (*16S rRNA*) genes has been a standard in environmental microbiology, with Oxford Nanopore Technologies (ONT) offering advanced DNA sequencing to analyze fermented foods' biodiversity. This technology's long-read capabilities enhance understanding of microbial populations and fermentation processes, allowing better production control through real-time monitoring. The full-length *16S rRNA* sequences offer high taxonomic resolution, aiding in bacterial identification by covering all informative gene regions. NGS has been successfully applied to profile microbiota in various fermented foods worldwide, such as kefir [31], traditional fermented products [32], salted food [33], kimchi [34], budu [35], and masin [36]. This process allows for more research into how microbes interact and how they affect the taste and nutritional value of fermented foods.

Some reports show the microbiota diversity of dadih from Kampar, Riau, Indonesia, primarily through molecular methods. Exploration of microbial diversity found in fermented products, particularly food, can indicate fermented food quality. Therefore, this study aimed to explore the microbial diversity of dadih from Kampar, Riau, Indonesia. The NGS technology was used to collect data regarding the microbiota and biodiversity contained in dadih, which could be applied to improve the quality of existing local and traditional functional foods in the future. Analysis was also conducted to identify potential microbiota diversity in dadih, offering valuable information for dadih producers to improve the quality of their products and provide added value.

## Materials and Methods

### Ethical approval

This study has been conducted without the inclusion of live animals and ethical approval.

### Dadih sample preparation

**Figure 1.**
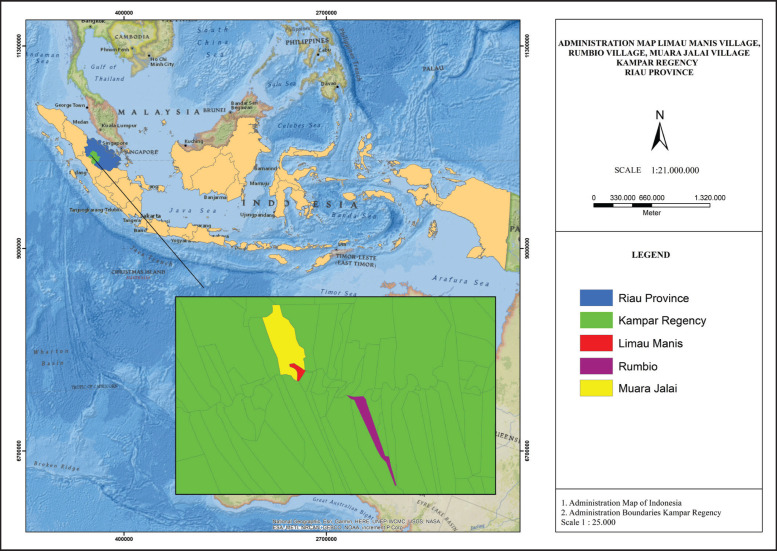
The location of dadih sampling in Kampar Regency, Riau, Indonesia.

Dadih samples were obtained from three producers in three different villages, namely Limau Manis (LM), Rumbio (RB), and Muaro Jalai (MJ), Kampar District, Riau Province, Indonesia (Fig. 1). Dadih samples were obtained from three producers in three different villages. Sampling was collected in duplicate at different production times for each producer. The dadih sample collected was stored at 4°C to avoid spoilage.

### Polymerase chain reaction amplification and NGS

Polymerase chain reaction amplification was carried out from the *16S rRNA* gene (V1-V9) region. This research uses primer sets 27F-1492R (27F 5'-AGA GTT TGA TCC TGG CTC AG-3' and 1492R 5'-GGT TAC CTT GTT ACG ACT T-3'). DNA concentration was determined using both NanoDrop spectrophotometers, which provide the long-read sequencing that covers the full-length sequence of the *16S rRNA* gene (V1-V9 regions), and a Qubit fluorometer. Library preparations were conducted using kits from Oxford Nanopore Technology. MinKNOW software version 24.04.5 was applied to operate the nanopore sequencing. Guppy version 6.5.7 with a high-accuracy model was used for base-calling [37]. NanoPlot was used to visualize the quality of the FASTQ data, and NanoFilt was used to filter the quality [38, 39]. A centrifuge classifier was used to classify the filtered readings [39]. The NCBI 16S RefSeq database (https://ftp.ncbi.nlm.nih.gov/refseq/TargetedLoci/) created the Bacteria and Archaea index. Downstream analysis and visualizations were performed using Pavian (https://github.com/fbreitwieser/pavian), Krona Tools (https://github.com/marbl/Krona), and RStudio with R version 4.2.3.

## Results and Discussion

### Diversity and composition of dadih microbiota

**Figure 2.**
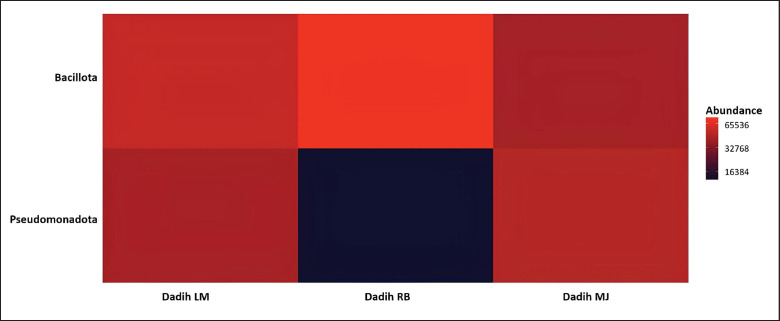
The dominant phylum in dadih from LM = Limau Manis, RB = Rumbio, and MJ = Muaro Jalai. The color degradation of each column shows the abundance of phyla in each Dadih.

Recent advancements in bioinformatics and metagenomic sequencing technologies have facilitated a deeper understanding of their microbial composition, metagenomic potential, and the diverse properties as well as beneficial activities of various fermented foods such as cheese [40], fermented sauce [41], and dadih, buffalo milk fermentation [42]. The results of the NGS analysis found two bacterial phyla, namely Bacillota and Pseudomonadota, in dadih produced by three different producers from different villages, with their biodiversity presented in Figure 2. Advancements in the taxonomic resolution of the fermented milk microbiome have significantly streamlined the identification and isolation of novel microbial resources [43] and recent technology [42].

**Figure 3.**
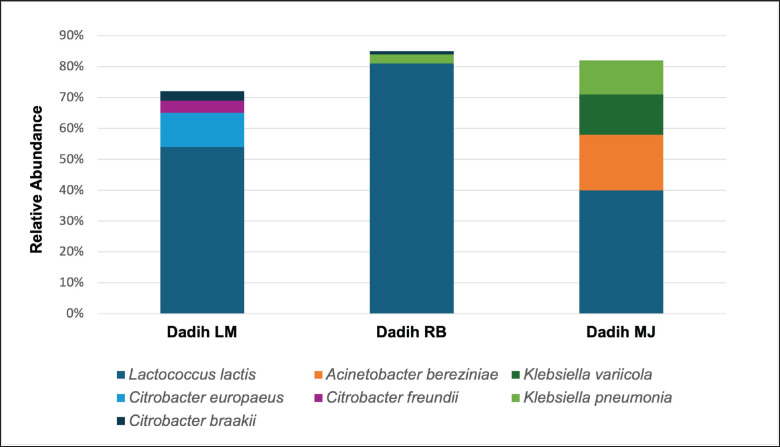
Distribution of bacterial species present in dadih produced from LM = Limau Manis, RB = Rumbio, and MJ = Muaro Jalai.

**Figure 4.**
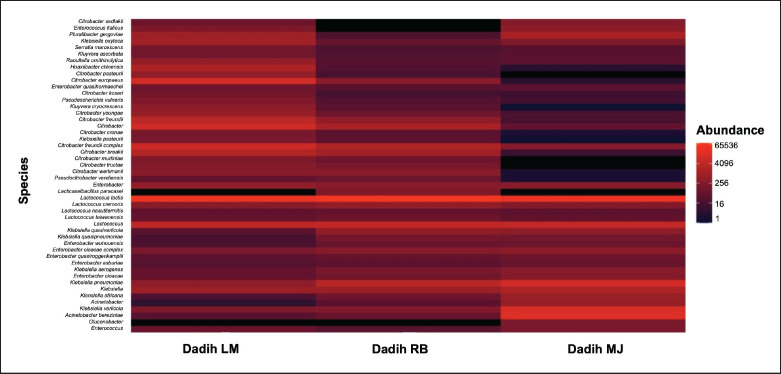
Heatmap species of dadih produced from LM = Limau Manis, RB = Rumbio, and MJ = Muaro Jalai.

The types of bacterial species in each dadih sample were different (Fig. 3). The total dominant microbial species in these Kampar dadih samples were *Lactococcus lactis*,* Acinetobacter bereziniae*,* Klebsiella variicola*, *Citrobacter europaeus*, *Citrobacter freundii*,* Klebsiella pneumoniae*, and *Citrobacter braakii. *Dadih microbiota was still dominated by *Lactococcus*, which was approximately 50% dominated by *Lactococcus.* *Lactobacillus* no longer dominated, although it was reclassified into 25 new genera in 2020. The heatmap species level showed that *L. lactis* still dominated the number of bacteria found in the three dadih samples, as indicated by a brighter color than the other species (Fig. 4).

**Figure 5.**
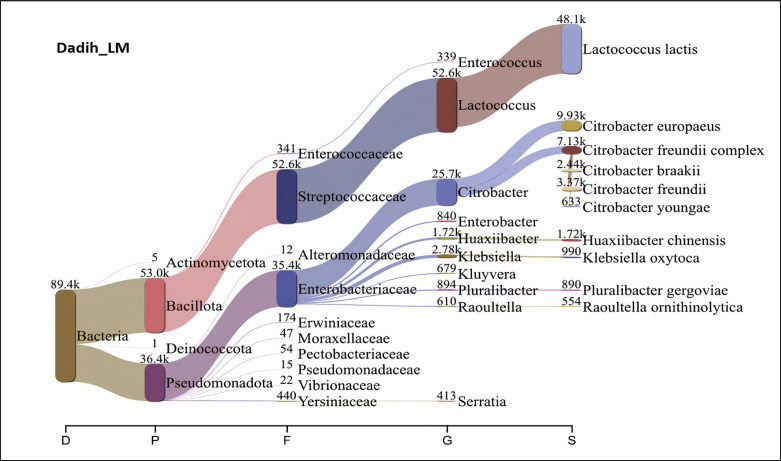
The relative proportion of phyla to species of dadih bacteria from Limau Manis village, Kampar district, Riau, Indonesia.

**Figure 6.**
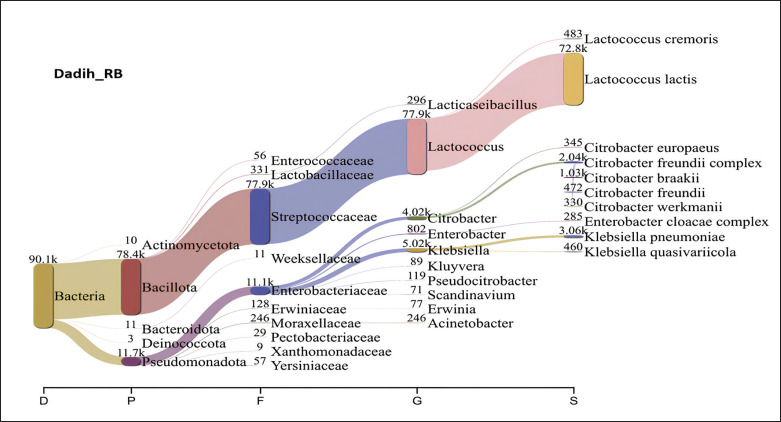
The relative proportion of phyla to species of dadih bacteria from Rumbio village, Kampar district, Riau, Indonesia.

**Figure 7.**
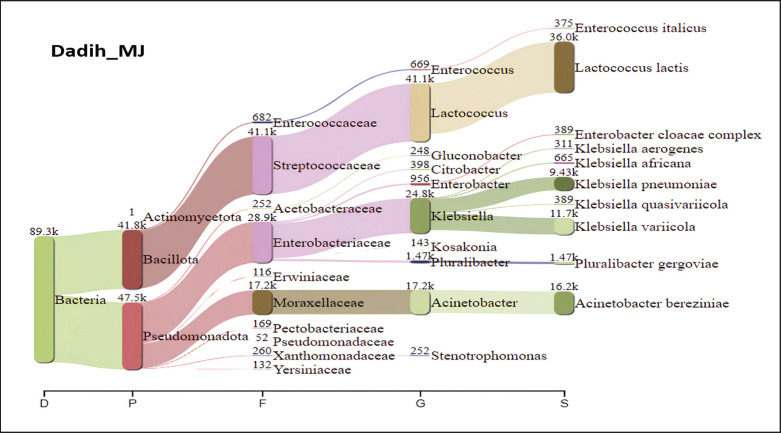
The relative proportion of phyla to species of dadih bacteria from Muaro Jalai village, Kampar district, Riau, Indonesia.

Figure 5 presents a Sankey diagram showing the microbiota of dadih from LM village, where the phylum Bacillota dominates with a total of 53.3 × 10^3^ (59.62%). Dadih LM was dominated by bacteria of the Streptococcaceae family (52.6 × 10^3^), the genus *Lactococcus* (52.6 × 10^3^), and the species *L*.* lactis* (48.1 × 10^3^). With an abundance of 72.8 × 10^3^, *L*.* lactis* also dominated the dadih from Rumbio village, accounting for 80% of all the bacteria (Fig. 6). The results of NGS dadih MJ (Fig. 7) showed that Pseudomonadota was the most common phylum (53.19%), but Streptococcaceae was the most common family (46.03%), which comes from the Bacillota phylum (46.71%). Bacteria species derived from the same phylum failed to match the high abundance of the phylum Pseudomonadota. In dadih MJ, the dominant bacterial species was *L*. *lactis* (40.31%), with a population of 36.0 × 10^3^. Based on Figures 5–7, the dominant species found in the three dadih was *L*.* lactis*, with an abundance of 53.80%, 80.80%, and 40.31% for dadih LM, RB, and MJ, respectively. The three villages' relatively similar ecosystems led to the highest relative abundance of the same dominant species. The results of the study of the dominant microbiota of dadih from Kampar, Riau, were *Lactococcus* (40.31%–80.80%), almost the same as that of dadih from West Sumatra, Indonesia, where *Lactococcus* (52%–83%) was analyzed from samples (*n *= 8) collected from four producers in different districts [44]. Farmers keep the same type of mud buffalo and use the same fermentation container, known as a gutter, for their buffaloes. Farmers in Riau and West Sumatra kept the same breed of mud buffalo and fed relatively the same 70%–80% roughage, although the type of bamboo for fermentation containers used was different. The two provinces (Riau and West Sumatra) were adjacent to each other, so the environment and weather are relatively similar.

**Figure 8.**
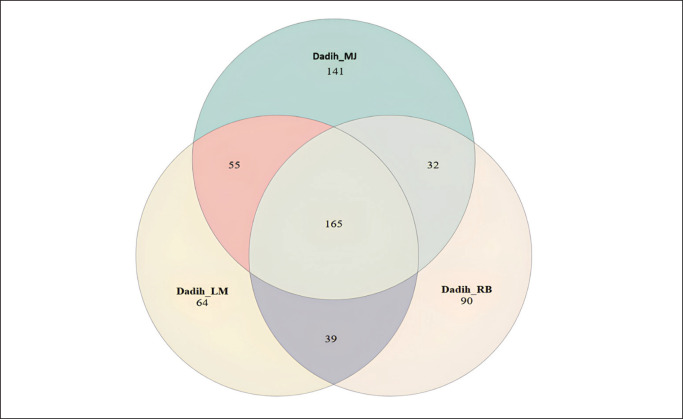
Venn diagram of the distribution of bacterial OTUs in dadih produced from LM = Limau Manis, RB = Rumbio, and MJ = Muaro Jalai.

**Figure 9.**
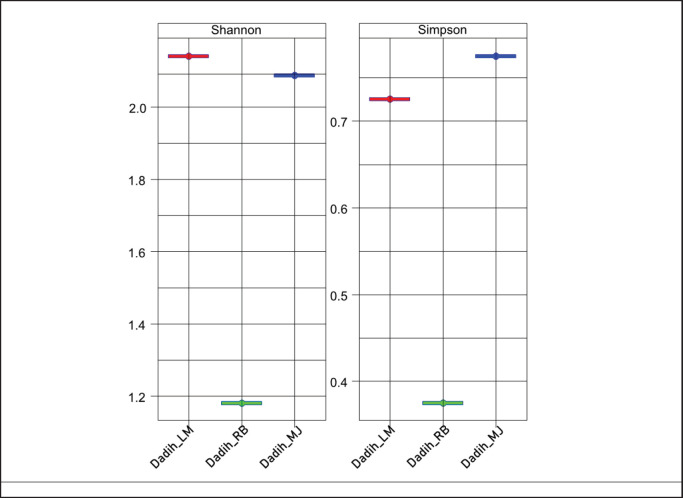
Evenness (Shannon) and richness (Simpson) of dadih produced from LM = Limau Manis, RB = Rumbio, and MJ = Muaro Jalai.

**Figure 10.**
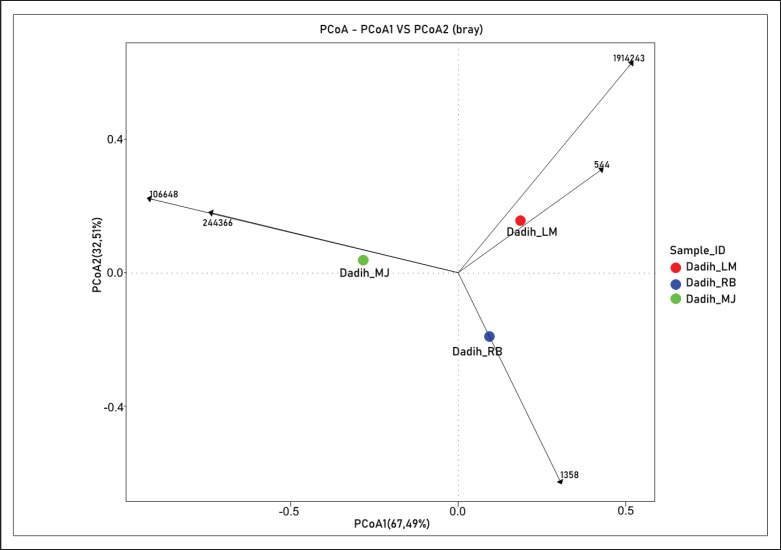
The PCoA of the weighted UniFrac (beta diversity) in the lactic acid bacteria of dadih.

### Similarity of the dadih microbiota of the area

The total number of bacteria species in each dadih sample was different. The total microbial species in dadih from MJ (393 species) was higher than those from RB (326 species) and LM (225 species). A Venn diagram showed the common and unique OTUs (operational taxonomic units) indicating the three villages for making dadih. The three dadih samples had 165 bacteria of the same species, and each dadih had different species of bacteria that the others did not have, with a total of 141, 90, and 64 species of bacteria found in the dadih from MJ, RB, and LM villages, respectively (Fig. 8).

### Alpha diversity of dadih microbiota

The alpha diversity of the microbial community in fermented food products has been studied using the metagenomic method [39, 40]. The metagenomic profiles of dadih from three villages at relatively different taxonomic and functional levels were generated. Based on the taxonomic analysis, alpha diversity estimates measuring the variability of species in the sample were first calculated, with the results shown in Figure 9. Greater microbial diversity was indicated by the higher values in terms of the number of rich species and the equilibrium between species abundance and evenness [41, 42]. Dadih LM had the highest Shannon value (~2.1), indicating the most extraordinary microbial diversity regarding the number of species and their even distribution. Dadih RB had a slightly lower Shannon value (~2.0) but still showed relatively high diversity, which approximately correlated with dadih LM. Meanwhile, dadih MJ had the lowest Shannon value (~1.2), indicating that microbial diversity was low. The low microbial diversity was due to the location of the three villages in the same district and the type of milk-producing buffalo and feed given. Bamboo tubes used for fermentation were relatively similar. Taxonomic features of fermentation products are primarily determined by the substrate being fermented [45].

Simpson's index values close to 1 show high diversity (more even species abundance), while values close to 0 indicate dominance by certain species. Dadih MJ had the highest Simpson's value (~0.8), indicating a relatively even abundance of species. Dadih LM also had a high Simpson's value (~0.75), showing similar conditions to dadih MJ. Meanwhile, Dadih RB had the lowest Simpson's value (~0.4), confirming that the microbiota tended to be dominated by certain species with a less even distribution.

### Beta diversity of dadih microbiota

Variations between samples or groups were measured using beta diversity. Figure 10 shows beta diversity measurements organized according to Bray–Curtis dissimilarity using a principal coordinate analysis (PCoA) layout. The PCoA data formed separate visible clusters on the graph, indicating that the microbiota from the village had a similar composition internally but differed from others. The same results [44] were also found in dadih produced in other locations in West Sumatra Province, where the microbiota had almost the same composition in one region. Based on this study, the microbiota in the three dadih samples were relatively similar.

## Conclusion

In conclusion, metagenomic analysis of dadih from producers in different villages showed that the bacterial diversity was hardly different. Dadih MJ had the highest number of microbial species (393), followed by RB and LM with 326 and 225, respectively. In all villages, the same 165 bacterial species were found, predominantly consisting of *L. lactis*. Dadih LM had the most diverse microbiota, with a more significant number of species and an even abundance distribution. These results suggested a possible fermentation environment or traditional practices that favored microbial diversity. Dadih RB also showed high diversity, although slightly below dadih LM. Dadih MJ had the lowest microbiota diversity, indicating dominance by certain species.

## List of Abbreviations

DNA, deoxy-nucleic acid; GABA, γ-aminobutyric acid; LAB, lactic acid bacteria; LDL, low-density lipoprotein; LM, Limau Manis; MJ, Muaro Jalai; NGS, next-generation sequencing; PcoA, Principal Coordinate Analysis; PCR, polymerase chain reaction; RB, Rumbio; rRNA, ribosome-ribonucleic acid.
